# Palliative Care Declarations: Mapping a New Form of Intervention

**DOI:** 10.1016/j.jpainsymman.2016.05.009

**Published:** 2016-09

**Authors:** Hamilton Inbadas, Shahaduz Zaman, Alexander Whitelaw, David Clark

**Affiliations:** School of Interdisciplinary Studies, University of Glasgow, Glasgow, United Kingdom

To the Editor:

It is 21 years since the *JPSM* published the Declaration of Florianópolis, drawing attention to the need for improved access to pain and palliative care services in Latin America.^1^ In the intervening years, there has been a growing tendency for palliative care associations and organizations to issue formal public statements of this type. Declarations have become part of the international palliative care landscape. They appear to require significant orchestration and planning, and yet they have not been examined from a research perspective. Defined as “statement(s) of intent or summaries of the desirable situation to which participants intend to work and to which they would like to encourage others to work,”^2^ declarations highlight matters of particular concern or call others to action in some way. They are a window on the priorities emerging in the field.

Understanding why, how, and with what effect declarations are produced has the potential to inform those who develop them and to improve their formulation and impact in the future. Our exploratory study set out to 1) map the emergence of the practice of palliative and end-of-life care declarations in the international context, 2) capture their form and characteristics, and 3) assess what is known about their purpose. To achieve this, we built a comprehensive collection and timeline of declarations that relate to palliative and end-of-life care, and are available in the public domain.

## Methods

Palliative and end-of-life care declarations published in the English language were collected using a four-stage approach. Our method was systematic but inevitably had some ad hoc elements, given the undocumented terrain we were investigating. Systematic searches on the Internet using the key words: palliative care, end-of-life care, declaration, manifesto, charter, commitment, and proclamation yielded 22 declarations. Web site scrutiny, especially the advocacy pages, of palliative care associations and organizations yielded seven declarations. A social media appeal through a blog post (by H.I.) listing the examples already found and requesting details of others was made, yielding one declaration. Finally, monitoring of social media from March 2015 to February 2016 led to the identification of four more declarations.

A timeline of palliative and end-of-life care declarations was created, and content analysis was undertaken to identify the geographical scope, relevant organizations, format of the documents, and key issues addressed.

## Results

### Timeline

Thirty-four palliative care declarations were identified in the period 1983 to February 2016 (Table 1). The timeline suggests a progressive increase in the production of declarations with 16 declarations published in the five-year period 2011 to 2015.

### Geographical Scope

The declarations were found to differ in their intended geographical reach. Seventeen were global in ambition. Nine declarations were international in focus but restricted to a particular world region or set of countries (two each for Latin America, Europe, and the developing countries and one each for Eastern Europe, Sub-Saharan Africa, and selected countries from Europe). Of the six declarations with national scope, three were issued in England by the National Council for Palliative Care and relate specifically to the U.K. general elections of 2005, 2010, and 2015.^3^ Two declarations had a specifically regional focus within a country—the province of Ontario, Canada, and the state of Kerala, India.

### Key Organizations

Palliative care associations dominate the production of the declarations, followed by associations from other fields of medicine, human rights organizations, academic institutions, and charitable organizations. The International Association of Hospice and Palliative Care, the European Association for Palliative Care, and the Worldwide Hospice Palliative Care Alliance are the organizations involved in the largest number of declarations.

Most organizations and associations publishing palliative care declarations are based in Europe. India and China are the only Asian countries that had organizations involved in palliative care declarations. In addition to issuing their own declarations, palliative care associations from Canada also were found to be engaging in local collaborations in issuing declarations of partnership. The U.S. was notably absent from the production of palliative declarations, being represented only through the engagement of the two global human rights organizations, which are U.S. based.

### Formats

The documents take different formats, revealing their varied purposes. Some set out “recommendations” about palliative care services, education, training provision, or policy changes. Others enshrine a clear “call to action” where the target audience can be government or the palliative care community itself. Some detail “statements of convictions” from the representing organizations, some outline specific “action plans” that the associations and their members seek to undertake, some harness the commitment of the producing organizations, and some draw attention to specific topics through a “description of issues” relevant to palliative care. Many declarations contain more than one of these formats.

### Key Issues

Content analysis of the 34 declarations showed that most draw attention to more than one issue. The need for “palliative care education” was highlighted most frequently, followed by demands for policy change, advocacy for better palliative care provision, and the need for pain relief. Considering the entries for pain relief, drug availability, and opioid availability as a single group, the demand for pain relief and symptom control is the concern most often raised in the declarations. Other issues highlighted are the need for palliative care research, building public awareness, the recognition of palliative care as a human right, and the benefits of a multidisciplinary approach to palliative care. Some call attention to children's palliative care.

## Comment

The practice of producing palliative care declarations has become a significant feature of the field of palliative care over the past three decades. Yet very little commentary or analysis exists on the process of making and disseminating palliative care declarations. The Budapest Commitments, which have led to further publications, include updates on progress and have a dedicated Web page on the context, process of development, and progress (2007–2011).^4^, ^5^ More of this approach is needed to better understand the value of declarations. Some commentators also refer to declarations when representing the voice of the palliative care community and promoting palliative care as a human right.^6^

The World Health Assembly Resolution of 2014 can arguably be considered the highest level advocacy document among all the declarations identified. However, only two of the seven declarations published after the World Health Assembly Resolution make reference to it or build on its recommendations.

Palliative care declarations seem to follow some well-established advocacy principles: starting with agenda setting, gathering relevant information, consideration of potential solutions, and preparation of recommendations for action or policy change.^7^, ^8^, ^9^ Monitoring the impact and ongoing improvement of strategies is recommended as part of implementing palliative care advocacy.^2^ Such assessment is lacking with regard to palliative care declarations. Further studies are needed to understand the process of their formation and their impact.

This exploratory study has led to the identification of the following research questions: 1) How and with what intentions are palliative care declarations developed? 2) What is the influence of palliative care declarations on the global development of palliative care? and 3) What measures are needed for an effective assessment of the impact of individual palliative care declarations? Addressing these research questions would enrich the understanding of the role of declarations as advocacy interventions in the global palliative care context.

## Figures and Tables

**Table 1:** Thirty-Four Palliative Care “Declarations”: 1983 to February 2016^a^

Year	Name of Declaration and Geographical Scope	Source	Recommendations and Key Content
1983	Declaration of Venice on terminal illness (Global)	Macpherson G. World Medical Association in Venice: BMA fails to reform constitution. *Br Med J* (Clin Res Ed). 1983;287:1644.	• The physician may relieve suffering of a terminally ill patient by withholding treatment • Withholding treatment does not free the physician from the obligation to assist the dying person and give necessary medications • The physician may refrain from using any extraordinary means that would prove of no benefit for the patient.
1994	The Declaration of Florianópolis (Latin America)	Stjernsward J, Bruera E, Joranson D, et al. Opioid availability in Latin America: the declaration of Florianopolis. *J Pain Symptom Manage*. 1995;10:233–236.	• The WHO should report patterns of use of opioids • Members to work with respective health ministries • Make available advice on legislation • Encourage multinational companies to bring in opioids • Encourage national companies to produce opioids at lower cost
1995	Barcelona Declaration on Palliative Care (Developing countries)	Barcelona Declaration on Palliative Care. *EJPC* 3 (1) 15.	• Develop clear informed policies • Implementation of specific services • Education of health professionals • Make necessary drugs available
1998	The Poznan Declaration (Eastern Europe)	The Poznan Declaration. *EJPC* 6 (2) 61–65.	• Promote national policies, education, and drug availability • Develop multidisciplinary palliative care services • Build wider awareness
2002	Cape Town Declaration (Sub-Saharan Africa)	Mpanga Sebuyira L, Mwangi-Powell F, Pereira J, Spence C. The Cape Town palliative care declaration: home-grown solutions for sub-Saharan Africa. *J Palliat Med* 2003;6:341–343.	• Palliative care is a right for everyone • Appropriate drugs should be made available • Education programmes should be established • Palliative care should be provided across all levels of care
2004	Charter for the Normalization of Death, Dying and Loss (Global)	Silverman P. The 2004 Tucson IWG (International Work Group): Charter for the Normalization of Dying, Death and Loss. OMEGA-*J Death Dying* 2005;50:331–336.	• Advocacy to recognize death as normal human experience • Involvement and partnerships with community • Political lobbying • Target legislative changes
2004	Palliative Care Manifesto (UK)	http://www.politicsresources.net/area/uk/ge05/man/groups/PalliativeCareManifesto.pdf	• Proposes additional £100 million annual investment in palliative care • Proposes introduction of monitoring care of the dying • Proposes a national training programme in palliative care
2005	Korea Declaration on Hospice and Palliative Care (Global)	http://hospicecare.com/uploads/2011/8/Korea_Declaration.pdf	• Include hospice and palliative care in government health policies • Access to hospice and palliative care is a human right • Integrate hospice and palliative care education and training into undergraduate and postgraduate curricula of medicine, nursing, research, and other disciplines • Make necessary drugs available, including affordable and available morphine to the poorest • Make hospice and palliative care available to all citizens
2006	WMA Resolution of Venice on Terminal Illness (Global)	http://www.wma.net/en/30publications/10policies/i2/	• Physicians should recognize the right of patients to develop written advance directives • Physicians should ensure psychological and spiritual resources are available • National Medical Associations should encourage governments to invest additional resources for palliative care and should advocate for a network of palliative care institutions/organisations • Medical schools' curricula should include palliative care
2006	The Declaration of Venice: palliative care research in developing countries (Developing Countries)	http://hospicecare.com/about-iahpc/contributions/venice-declaration/english-declaration/	• Invite academic institutions to ensure palliative care research • Governments to support palliative care research • Institutions to learn from existing successful collaborative palliative care research initiatives
2007	Budapest Commitments (Global)	http://www.eapcnet.eu/Themes/Policy/Budapestcommitments.aspx	• Ensure availability and access to all palliative care essential medicines • Increase the rational use of opioids • Produce a report on the state of development and present to national authorities • Have palliative care inserted in the curriculum for medical/nursing students • Define standards of care • Incorporate proposals presented in the Venice Declaration to support the development of research in palliative care
2008	International Children's Palliative Care Network Charter (Global)	http://www.icpcn.org/icpcn-charter/	• Every child should expect individualized, culturally, and age-appropriate palliative care, begun at the time of diagnosis and continued alongside any curative treatments throughout the child's illness, during death, and in bereavement • The child's parents or legal guardians should be full partners in all care and decisions • The child shall be encouraged to participate in decisions • A sensitive, honest approach will be the basis of all communication • The child will have access to education and wherever possible be provided with opportunities to play • The child will have access to leisure opportunities and interaction with siblings and friends and participation in normal childhood activities • The child will have an opportunity to consult with a pediatric specialist • The child and the family shall be entitled to a named and accessible key worker • The child's home shall remain the center of care whenever possible • The child and family members, including siblings, shall receive culturally appropriate, clinical, emotional, psychosocial, and spiritual support • Bereavement support for the child's family shall be available for as long as it is required
2008	Panama Proclamation (Latin America)	http://hospicecare.com/uploads/2011/8/panama_proclamation_pain_relief_as_a_human_right_english.pdf	• Member groups to promote pain relief and palliative care as a human right • The proclamation to be translated and promoted to governments • Copies of the proclamation to be sent to associates worldwide, including the United Nations and religious leaders worldwide
2009	Wuhan Declaration (China)	Qi M, Yuan C, Shukui Q, Guangru X, Jiejun W, Aiguo L, Jiliang Y, Hong Q, Yi C, Payne S, Shiying Y. Budapest commitments in China: from desire to action. *Eur J Palliative Care*. 2010; 17: 246–8.	• To include palliative medicine in clinical teaching programmes of undergraduates and in oncology modules and continuing education programmes • To explore the potential for developing a Chinese service provision model for cancer rehabilitation and palliative therapy • All oncology departments to supply at least two types of opioids and to draw up a list of basic drugs used in palliative care • Better training of health care professionals in the effective use of basic drugs for palliative therapy • Improved communication to better inform wider society
2009	IAHPC-WPCA joint declaration (Global)	http://hospicecare.com/uploads/2011/8/jdsc_en.pdf	• To work with governments and policy makers for the recognition of palliative care and pain treatment as fundamental human rights • Ensure availability of and access to opioids and other appropriate medication for the treatment of pain in adults and children • Ensure creation of positions in palliative care and pain treatment in academic institutions and support them with resources
2009	End-of-Life Care Manifesto 2010 (U.K.)	http://www.ncpc.org.uk/sites/default/files/2010Manifesto.pdf	• Ensure that the End of Life Care Strategy for England is fully implemented • Give strong political leadership and commitment • Put in place comprehensive out-of-hours services for palliative care • Ensure that training in palliative and end-of-life care is a core curriculum requirement • Equip people and the nation to become confident about discussing their wishes and priorities for end-of-life care, through supporting the awareness-raising activities
2010	Declaration on Palliative Care and MDR/XDR-TB (Global)	Connor S, Foley K, Harding R, Jaramillo E. Declaration on palliative care and MDR/XDR-TB. *Int J Tuberc Lung Dis*. 2012; 16: 712–713.	• Palliative care should be integrated alongside the prevention and treatment of MDR/XDR-TB • Palliative care should be integrated into the management of MDR/XDR-TB from diagnosis until the patient reaches cure or the end of life
2011	WMA Declaration on End-of-Life Medical Care (Global)	http://www.wma.net/en/30publications/10policies/e18/index.html.pdf?print-media-type&footer-right=%5bpage%5d/%5btoPage%5d	• Provide advance care planning to maintain patient dignity and freedom from distressing symptoms • Palliative care to be part of undergraduate and postgraduate education • Use palliative sedation proportional to situation but never intentionally to end life • More research needed to improve palliative care • National medical associations to develop policies on palliative care and palliative sedation • Recognize the needs of the family and children
2011	The Lisbon Challenge (Global)	http://www.eapcnet.eu/Themes/Policy/Lisbonchallenge.aspx	• National governments to check how well they perform with these objectives • Ensure access to essential medicines, including opioid medications, to all who need them • Develop health policies that address the needs of patients with life-limiting or terminal illnesses • Ensure that health care workers receive adequate training in palliative care and pain management at undergraduate levels • Ensure, through the development of structures and processes, the implementation of palliative care
2011	Declaration of Partnership and Commitment to Action (Ontario province, Canada)	http://health.gov.on.ca/en/public/programs/ltc/docs/palliative%20care_report.pdf	• Individuals and families to receive care and support through consultation and integrated delivery teams • Increase number of all types of professionals connected to the individual's care • Organizations collaborate on care plans • Individuals have advance care plans • Access to—and uptake of—education initiatives • Decrease in caregiver burden • Improved individual, caregiver, and provider experience • Improved pain and symptom management • Increase in the number of persons with advanced or end-of-life chronic disease receiving team-based care • Increase in the number of persons with advanced or end-of-life chronic disease discharged from hospital to team-based care • Change in the location of Ontario deaths
2011	OPCARE9 Liverpool Declaration (UK, Germany, The Netherlands, Italy, Sweden, Slovenia, Switzerland, Argentina, New Zealand)	http://www.mcpcil.org.uk/media/Doc%204%20OPCARE9%20Report.pdf	• Improve societal and public health approaches • Improve health care structures • Implement curricula in health care and volunteer education • Improve conditions for research
2011	Lucknow Declaration/Palliative Care Declaration (India)	http://canceraidsocietyindia.org/palliative-care/palliative-care-declaration/	• Increase the number of states that simplify opioid legislation and make pain relief and palliative care an essential service in all the cancer treatment institutions and government hospitals along with home-based care, including access to opioids such as oral morphine, symptom control, psychological, and family support • Intensive education on palliative care for health care professionals and inclusion in nursing, undergraduate, and postgraduate medical curricula • Advocacy and mass sensitization about the need for palliative care • Freedom from pain should be regarded a human right
2012	Manifesto—Better Palliative Care for Older People (Europe)	http://www.eapcnet.eu/LinkClick.aspx?fileticket=Oy94klBm_dA%3D&tabid=1854	• Recognize that older people with chronic diseases have the right to the best possible palliative care approach • Promote public awareness • Promote collaborative effort between geriatric and palliative medicine • Invest in education • Invest in research • Create an EU platform for the exchange, comparison, and benchmarking of best practices
2013	The Prague Charter (Global)	http://hospicecare.com/uploads/2013/6/PragueCharterPetition.pdf	• Call on governments to develop comprehensive health care policies that provide integrated palliative care • Make available essential medicines and opioids • Include support to relatives • Ensure health care workers receive training in palliative care and pain management • Motivate primary health care professionals to integrate palliative care in their services
2013	The Charter for the Rights of the Dying Child (Global)	http://www.maruzza.org/en/wp-content/uploads/2014/12/CartaDiTrieste200x240_ingleseUNICO.pdf	• To be considered a person until death irrespective of age, location, illness, and care setting • To receive effective treatment for pain and physical and psychological symptoms • To be listened to and properly informed about his or her illness • To participate in care choices about his or her life, illness, and death • To express and, whenever possible, have his or her feelings, wishes, and expectations taken into account • To have his or her cultural, spiritual, and religious beliefs respected and receive spiritual care and support in accordance with his or her wishes and choices • To have a social and relational life suitable to his or her age, illness, and expectations • To be surrounded by family members and loved ones who are adequately supported and protected from the burden of the child's illness • To be cared for in a setting appropriate for his or her age, needs, and wishes and that allows the proximity of the family • To have access to child-specific palliative care programmes that avoid futile or excessively burdensome practices and therapeutic abandonment
2014	Mumbai Declaration (Global)	http://palliativecare.in/mumbai-declaration/	• Children have the right to high-quality palliative care • Euthanasia is not part of children's palliative care and is not an alternative to palliative care • Governments to transform children's lives through the development of and access to children's palliative care, appropriate pain, and symptom management and by supporting children and their families
2014	WHO: World Health Assembly Resolution (Global)	http://www.thewhpca.org/resources/item/palliative-care-resolution-providing-comprehensive-care	• Member states to develop, strengthen, and implement, where appropriate, palliative care policies to integrate palliative care at all levels of healthcare • To ensure adequate domestic funding and allocation of human resources • To include palliative care as an integral component of ongoing education • Undertake palliative care need assessment, including pain management medication requirements
2014	Manifesto—The crisis facing terminally ill people and their families (UK)	http://www.palliativecare2020.eu/declaration/	• Make a commitment to introduce 24/7 care, advice, and support for terminally ill people and their families • Make social care free and fast for terminally ill people and their families • Accelerate co-ordination between services • Increase medical research budget for developing better ways of caring for terminally ill people and their families • Improve data collection for better care
2014	Montreal Declaration on Hospice and Palliative Care (Global)	http://www.palliative.ch/fileadmin/user_upload/palliative/publikum/2_PalliativeCare/Montreal_Declaration_on_Hospice_Pallitive_Care.pdf	• Inclusion of hospice and palliative care in the United Nations Sustainable Development Goals
2014	European Declaration on Palliative Care (Europe)	http://www.palliativecare2020.eu/declaration/	• Recognize high-quality palliative care is a public health priority • National and international health care policies to include palliative care as an essential component • Ensure access to specialist multidisciplinary palliative care • Promote a paradigm shift in health and social care toward basic palliative care skills for all health care workers • Invest in curriculum development and education in palliative care across all disciplines of health at undergraduate and postgraduate level • Establish palliative care as a speciality • Provide education of the public and training of volunteers • Increase funding opportunities for national and international research in palliative care
2015	Declaration by the People of Kerala (Kerala, India)	http://palliumindia.org/cms/wp-content/uploads/2015/02/Declaration-by-the-People-of-Kerala-2-Feb-2015.pdf	• The Kerala Government to direct all hospitals in the state to stock and dispense morphine, the affordable “essential medicine” on presentation of a correct prescription • All hospitals to have at least one doctor and nurse trained in pain management and palliative care on staff • Hospitals in Kerala to develop appropriate end-of-life care policies that respect the dignity of the individual, relieve suffering whenever possible, and facilitate end-of-life care in the presence of the family avoiding inappropriate and expensive interventions • Direct public health and community organizations to provide professional and volunteer training in crucial conversations on topics such as end-of-life care, disposition of assets, living wills, and the right to refuse artificial life-support measures in the face of clinical opinion when cure is no longer an option, and further treatment is futile
2015	Compassionate Cities Charter (Global)	http://www.ncpc.org.uk/sites/default/files/Public_Health_Approaches_To_End_of_Life_Care_Toolkit_WEB.pdf	• Schools, workplaces, and trade unions to have annually reviewed policies or guidance documents for dying, death, loss and care • Churches and temples to have dedicated groups for end-of-life support • Hospices and nursing homes to have community development programmes • Create incentives for compassionate organizations • Publicize policy, service, and funding information
2015	Religions of the World Charter for Children's Palliative Care (Global)	http://www.maruzza.org/en/wp-content/uploads/2015/11/Charter-Text.pdf	• To affirm the essential right of all seriously ill children and their families to receive palliative care appropriate for children • To call for the broadest possible dissemination of children's palliative care.
2016	Pune Declaration (India)	http://palliativecare.in/pune-declaration/	• Deliver adequate funding and effective implementation to the National Programme for Palliative Care • Establish a rightful place for palliative care in non-communicable diseases control programme • Implement the amendment of the Narcotic Drugs and Psychotropic Substances Amendment Act of 2014 • Promote undergraduate palliative care education

EU = Europeran Union; WHO = World Health Organization; MDR/XDR-TB = multidrug-resistant/extensively drug-resistant tuberculosis.

a Some declarations are not specific to palliative care. These were included because they deal with end life care issues. The Declaration of Florianópolis is specific to opioid availability. However, it came out of a palliative care context and seeks to improve opioid availability for use in palliative care.
